# The Role of Short-Chain Conjugated Poly-(*R*)-3-Hydroxybutyrate (cPHB) in Protein Folding

**DOI:** 10.3390/ijms140610727

**Published:** 2013-05-23

**Authors:** Rosetta N. Reusch

**Affiliations:** Department of Microbiology and Molecular Genetics, Michigan State University, East Lansing, MI 48824, USA; E-Mail: rnreusch@msu.edu; Tel.: +1-517-349-3544; Fax: +1-517-353-1793

**Keywords:** poly-(*R*)-3-hydroxybutyrate, cPHB, PHBylation, protein-folding, protein-targeting

## Abstract

Poly-(*R*)-3-hydroxybutyrate (PHB), a linear polymer of *R*-3-hydroxybutyrate (*R*-3HB), is a fundamental constituent of biological cells. Certain prokaryotes accumulate PHB of very high molecular weight (10,000 to >1,000,000 residues), which is segregated within granular deposits in the cytoplasm; however, all prokaryotes and all eukaryotes synthesize PHB of medium-chain length (~100–200 residues) which resides within lipid bilayers or lipid vesicles, and PHB of short-chain length (<12 residues) which is conjugated to proteins (cPHB), primarily proteins in membranes and organelles. The physical properties of cPHB indicate it plays important roles in the targeting and folding of cPHB-proteins. Here we review the occurrence, physical properties and molecular characteristics of cPHB, and discuss its influence on the folding and structure of outer membrane protein A (OmpA) of *Escherichia coli*.

## 1. Introduction

**Figure 1:**
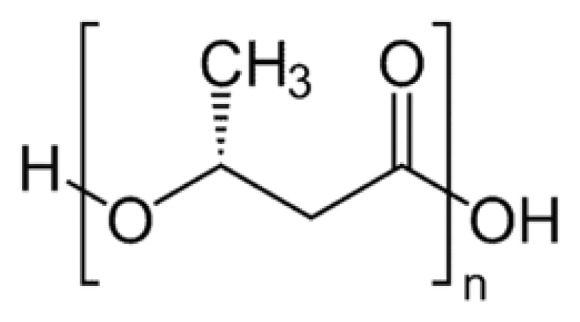
Chemical structure of poly-(*R*)-3-hydroxybutyrate (PHB).

**Figure 2:**
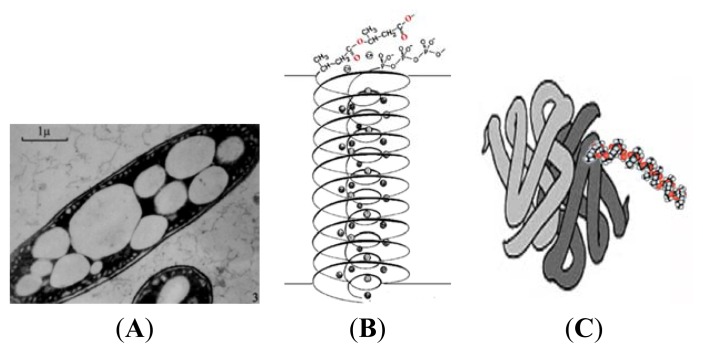
Classes of poly-(*R*)-3-hydroxybutrate. (**A**) Storage PHB. Ultrathin section of an *Azotobacter chrococcum* cell showing granules of PHB surrounded by a membrane (X 56,200) From Nuti *et al.* [4]; (**B**) Medium-chain length PHB. Sketch of PHB molecule surrounding and noncovalently associated with a molecule of calcium polyphosphate in the bilayer; (**C**) Short-chain PHB covalently attached (cPHB) to protein (grey).

Poly-(*R*)-3-hydroxybutyrate (PHB), a linear polymer of *R*-3-hydroxybutyrate (R-3HB) (Figure 1), was first discovered in granular inclusion bodies within the cytoplasm of *Bacillus megaterium* in the mid 1920s by Maurice Lemoigne [1]. Similar PHB-containing granules were subsequently observed in other eubacteria and archaea, principally those that inhabit soil and water ecosystems [2–4] (Figure 2A). The granules frequently include some *R*-3-hydroxyvalerate and other hydroxy-acids, and thus they are often referred to as polyhydroxyalkanoates (PHA). The polymers in these complex subcellular organelles, known as carbanosomes, are very long-chain (10,000 to >1,000,000 residues), and are covered by a layer of lipids and proteins, which include enzymes involved in PHB synthesis and degradation [5–7]. PHB is accumulated by these prokaryotes when carbon sources are freely available but other nutrients are limited, thus PHB is considered to serve as a carbon and energy store in these organisms. The granules have attained considerable commercial importance as ingredients of biodegradable plastics. Recently, Elustondo *et al.* [8], using fluorescence spectroscopy, identified PHB-rich granules, which resembled bacterial carbanosomes and did not co-localize with mitochondria, lysosomes or endoplasmic reticulum, in the cytoplasm of cultured mammalian cells. It was suggested that the PHB may serve as a carbon and energy store for these cells. The identity of PHB was confirmed by a marked decrease in fluorescence intensity when the samples were treated with PHB depolymerase (PhaZ); however, the molecular weight of the polymer has not yet been determined.

PHB of medium-chain length was first discovered in the cytoplasmic membranes of genetically-competent bacteria by Reusch and Sadoff in 1983 [9]. Medium-chain PHB typically consists of 100–200 residues, but it may contain fewer residues due to the lability of ester bonds. Medium-chain PHB, like storage PHB, is insoluble in water and soluble in chloroform, and non-covalently associated with other molecules. The medium-chain length polyester was found in the membranes of competent bacteria which also accrue high molecular weight storage PHB—*Azotobacter vinelandii* and *Bacillus subtilis*—but it was also unexpectedly present in the membranes of competent bacteria—*Haemophilus influenzae* and *Escherichia coli*—which do not accumulate PHB granules [9,10]. In 1989, medium-chain length PHB was recovered from membranes, mitochondria and microsomes of eukaryotes [11], and in 1992 from lipoproteins (very low density and low density (VLDL and LDL) of human plasma [12]. The identity of medium-chain length PHB in representative prokaryotic and eukaryotic organisms was confirmed by ^1^H-NMR spectroscopy by Reusch [13] in 1992 and corroborated by Seebach *et al.* [14] in 1994. Medium-chain length PHB has been found associated with inorganic polyphosphate in noncovalent complexes (Figure 2B) that are postulated to play a role in transbilayer transport of cations [15–17] and deoxyribonucleic acids [18–20].

In 1996, Huang and Reusch [21] discovered short-chain PHB (≤10 residues) covalently bound to specific proteins in the membranes and the cytoplasm of *E. coli* cells (Figure 2C). This short-chain conjugated PHB has been termed cPHB. It is generally thought to consist of <20 residues but this remains unclear due to the lability of the ester bond and the paucity of samples examined to date. Unlike storage PHB that is segregated within cytoplasmic granules and medium-chain PHB that is “dissolved” in lipid environments, cPHB has been found in all cell compartments of prokaryotes and eukaryotes and in intracellular fluids [22–28]. Indeed, the majority of PHB in cells that do not accumulate long-chain storage PHB is cPHB. Significantly, unlike storage PHB and medium-chain length PHB, cPHB cannot be isolated from cells or organelles by extraction with chloroform. The ubiquitous occurrence of the medium-chain and short-chain polyester, suggests that PHB, like polyisoprenoids, polypeptides, polysaccharides, and polynucleotides, is a fundamental constituent of biological cells. Since its synthesis requires only acetate and reducing potential, both of which were available in the primordial soup, the structurally simple PHB may have been the earliest of the biological organic polymers [21,29].

## 2. Synthesis of PHB

**Figure 3:**
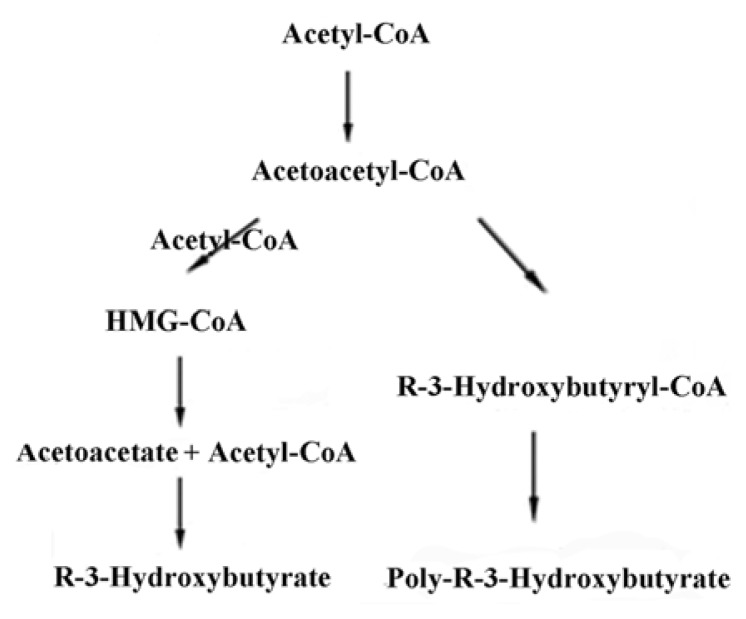
Biosynthetic pathways for PHB metabolites in prokaryotes and eukaryotes. In all organisms, acetoacetyl-CoA is formed by condensation of two acetyl-CoA. In prokaryotes (**right**), acetoacetyl-CoA is reduced by NADPH to *R*-3-hydroxybutyryl-CoA, which is then polymerized to PHB. In eukaryotes (**left**), a third acetyl-CoA condenses with acetoacetyl-CoA to form 3-hydroxy-3-methylglutaryl-CoA (HMG-CoA), which is subsequently decomposed to acetoacetate and acetate. Acetoacetate is then reduced by NADH to *R*-3-hydroxybutyrate.

*R*-3-hydroxybutyrate (R-3HB) is a well-known metabolite in fatty acid synthesis in both prokaryotes and eukaryotes [2,3,30]. The synthesis of *R*-3HB begins with the condensation of two molecules of acetyl-CoA to form acetoacetyl-CoA by a ketothiolase enzyme. In prokaryotes, this intermediate is subsequently reduced with NADPH to *R*-3-HB-CoA by acetoacetyl-CoA reductase, and *R*-3-HB-CoA may then be polymerized to form PHB by the enzyme PHB synthase (Figure 3). In eukaryotes, 3-hydroxy-3-methylglutaryl-CoA synthase (HMG synthase) catalyzes the condensation of acetoacetyl-CoA with a third acetyl-CoA to form 3-hydroxy-3-methylglutaryl-CoA (HMG-CoA). The enzyme HMG-lyase then catalyzes the decomposition of HMG-CoA to form acetoacetate and acetyl-CoA, and acetoacetate is further reduced with NADH by *R*-3-HB dehydrogenase to form *R*-3-HB (Figure 3). Enzymes that polymerize *R*-3-hydroxybutyrate or its CoA ester have not yet been identified in eukaryotes.

## 3. Physical Properties of PHB

PHB consisting of more than six residues is insoluble in water and soluble in chloroform [2]. The PHB molecule is amphiphilic—hydrophobic methyl groups alternate with hydrophilic carbonyl ester groups (Figure 1); accordingly, the methyl groups may engage in hydrophobic interactions and the ester carbonyl oxygens may serve as hydrogen-bond acceptor or as ligands for coordinate bonds to cations. Moreover, polyesters such as PHB, in contrast to polyamides and polypeptides, have highly flexible backbone structures, since they lack the stabilizing element of internal hydrogen bonds and the rigidity of a peptide bond. The extremely high flexibility of the PHB backbone at physiological temperatures has been amply demonstrated by Seebach *et al.*, using circular dichroism and fluorescence (FRET) measurements [31], nuclear magnetic resonance (NMR) spectroscopy [32,33] and molecular dynamics simulations [34]. However, it is important to note that the glass temperature of PHB is ~10 °C, so that the PHB backbone becomes increasingly more rigid as temperatures are lowered below the physiological range.

PHB also has uncommon solvating capacity. It may be unique among biological polymers in having the structural features common to a small group of synthetic polymers, known as polymer electrolytes [35–37] that are distinguished by their ability to “dissolve” salts. Significant characteristics of this class of polymers are (1) flexible backbones with low barriers to bond rotation to ease segmental motions of the polymer chain; (2) heteroatoms, such as ester carbonyl oxygens, that have sufficient electron donor power to form coordinate bonds with cations; (3) a suitable distance between the electron donating groups to permit the formation of multiple intra-polymer coordinate bonds to cations. The stability of such polymer salt complexes is enhanced by the entropic advantage known as the “polymer effect”, attributable to the cooperative effect of neighboring ligands attached to a common backbone.

PHB “dissolves” salts by encircling them and replacing the water of hydration surrounding the cation with coordinate bonds to its ester carbonyl oxygens. The ability of PHB to solvate salts is however limited. Ester carbonyl oxygens are weak Lewis bases of low polarity and hence PHB form coordinate bonds only with hard cations that have large solvation energies, such as the four major physiological cations, Na^+^, K^+^, Mg^2+^, and Ca^2+^. Moreover, as an aprotic polymer, PHB does not have hydrogen-bond donating groups that are needed to solvate anions [35–37]. Accordingly, PHB “dissolves” salts composed of hard cations and large anions with diffused charges that require little solvation, and may thereby serve to partition these salts into lipid bilayers or hydrophobic regions of proteins. This capacity of PHB has been well established experimentally. Seebach *et al.* [38] have found that the triolide of R-3HB forms crown ester complexes with alkali metals; Burger and Seebach [39] have shown that oligomers of R-3HB transport alkali and alkaline earth salts across methylene chloride layers in U-tubes; Seebach *et al.* [40] have demonstrated that PHB of 16 residues or multiples of 16 residues up to 96 residues form channels in planar lipid bilayers, and Fritz *et al.* [41] have described the gradient-driven transport of Ca^2+^ into liposomes by R-3HB oligomers.

## 4. cPHB-Proteins

It is increasingly apparent that cPHB-proteins are ubiquitous in biological cells, both prokaryotic and eukaryotic [8,11–14,28,42–44]. However, cPHB has remained largely unnoticed because, unlike the highly-visible phase-bright PHB granules, cPHB is sparse and obscure. Its exceptionally flexible backbone and lack of unusual atoms or functional groups makes cPHB nearly invisible in X-ray structures in which it may resemble detergent or lipid molecules. Whereas storage PHB and medium-chain length PHB are insoluble in water and soluble in chloroform, the solubility of a cPHB-protein depends on the nature of the protein itself and on the extent of cPHB modification. However, within a given protein, cPHB significantly alters the hydrophobicity of the protein segments to which it is attached and thereby influences their location within the mature protein.

**Figure 4:**
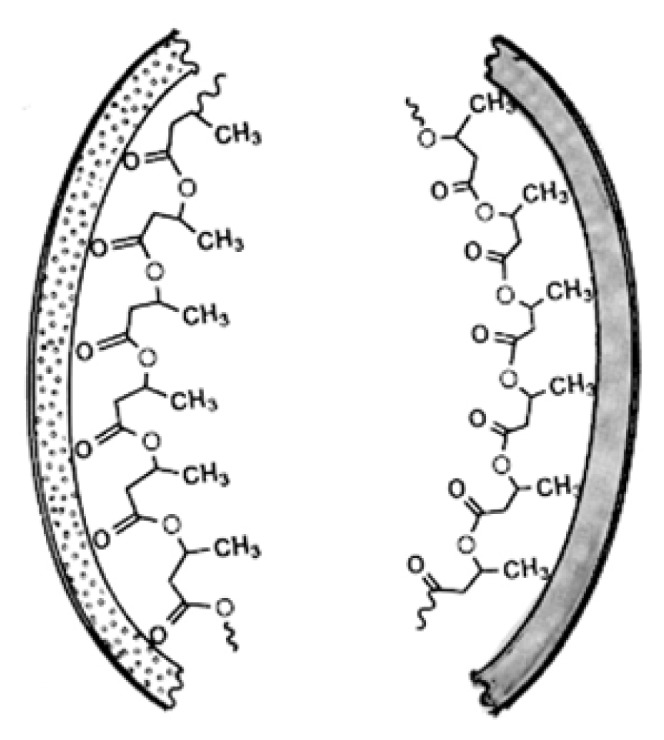
Sketch illustrating possible modes of noncovalent binding of cPHB to hydrophobic (shaded) and polar (dotted) region in polypeptides, thereby reversing their polarity. From Reusch and Gruhn, (1997) [44].

There are a number of ways in which the functional groups of cPHB may interact noncovalently with protein segments. Since the cPHB molecule is both highly flexible and amphiphilic, cPHB can adjust or reverse the polarity of peptide segments or act as an intermediary between polar segments and the bilayer (Figure 4) [44]. When the methyl groups of cPHB form hydrophobic bonds to nonpolar peptide residues, its ester carbonyl oxygens form a hydrophilic surface that may associate with polar molecules. Conversely, when the carbonyl oxygens of PHB act as hydrogen-bond acceptors or form coordinate bonds to bridging cations of polar peptides, its methyl groups form a hydrophobic surface that may promote movement of the peptide into the protein interior or assist its insertion into a phospholipid bilayer. Individually, such noncovalent bonds are weak, but the frequency at which the methyl groups and ester carbonyl oxygens repeat along the cPHB backbone allows each polymer molecule to have multiple interactions. The number and strength of these bonds would be determined by the primary structure and three-dimensional geometry of the protein at the binding site(s). The tenacity of the bonding, as evidenced by its ability to withstand heating in sodium lauryl sulfate, extraction with warm chloroform, and proteolysis, suggests that there is also a covalent bond between an amino acid residue and the terminal hydroxy group or S-CoA group of cPHB. This covalent attachment of cPHB is known as PHBylation.

Although the cPHB backbone is highly flexible at physiological temperatures, it becomes increasingly more rigid as temperatures approach the glass temperature. As a consequence, cPHB-modified proteins would fold very slowly or be unable to fold into their native conformations when maintained below physiological temperatures. The presence of cPHB may also render protein function(s) highly sensitive to temperature in this range. For example, Zakharian *et al.* [42,43] found that a member of the transient receptor potential (TRP) channel family of the melastatin subgroup, TRPM8, which is a major sensor for cold temperatures in the peripheral nervous system, is significantly modified by cPHB.

## 5. Identification of cPHB

**Figure 5:**
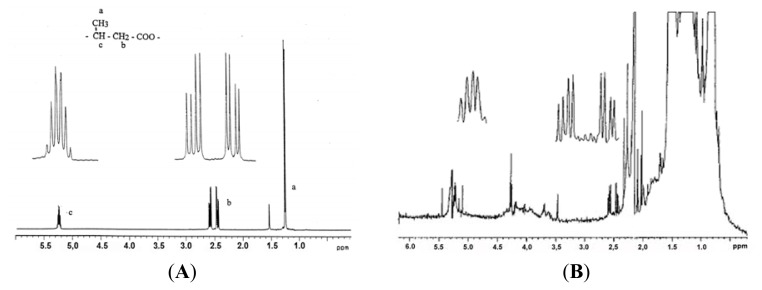
(**A**) ^1^H NMR spectra of PHB from *Alcaligenes*. From Reusch (1992) [13]; (**B**) ^1^H NMR spectra of the CHCl3:CH3OH (9:1) extract of the ribosomal fraction of *E. coli* JM101. The sample shows the characteristic methylene and methine protons of PHB; the methyl protons are hidden under resonances of impurities. Assignments: methylene protons split into an octet at 2.42–2.62 ppm, JAX 5.7, JBX 15.5; methane protons form a multiplet centered at 5.23 ppm. The assignments were confirmed by selective decoupling of the methine resonances. From Huang and Reusch 1996 [21].

**Figure 6:**
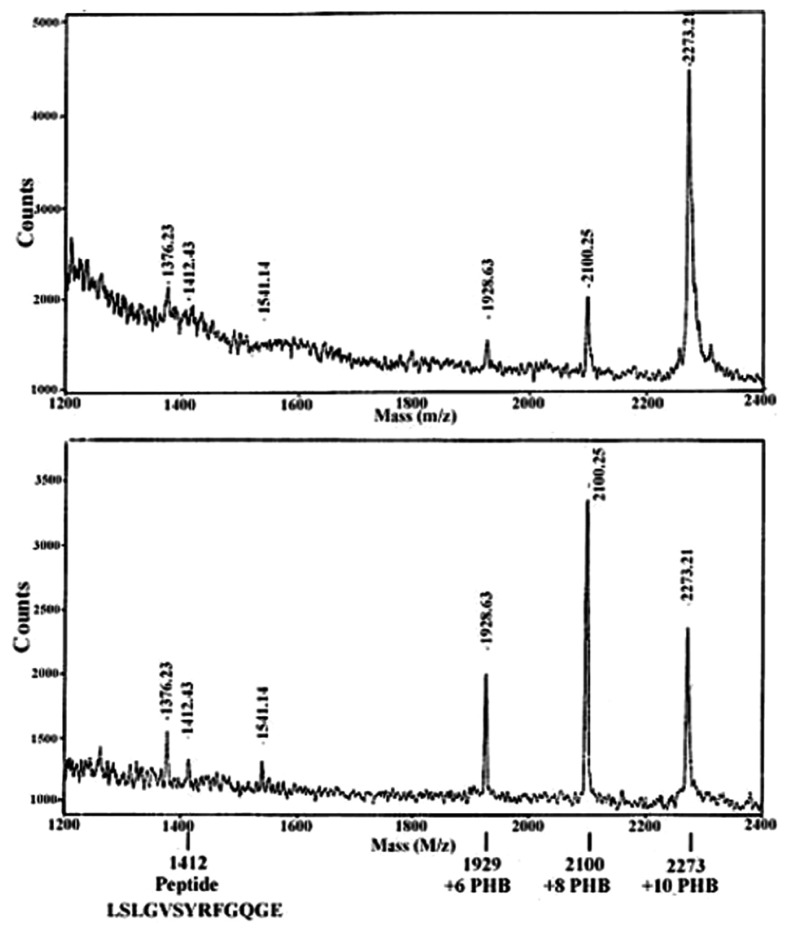
MALDI-MS of segment 162–174 of OmpA. Peptide LSLGVSYRFGQGE (162–174), obtained by cyanogen bromide and *Staphylococcus aureus* Glu-C digestion of wild-type OmpA. Matrix: a-cyano-4-hydroxycinnamic acid. MS analysis was performed using a Voyager Elite (Applied Biosystems) mass spectrometer in linear positive mode with a 50-ns delay. Laser strength was set to 1700 and low mass gate was set at 1000. Upper panel: initial spectrum. Lower panel: spectrum after ~3 additional seconds of the laser beam. From Xian *et al.* (2007) [26].

cPHB may be most easily and sensitively detected by Western blot analysis using anti-PHB IgG [44]. cPHB may also be detected and estimated by a chemical assay in which the polymer is converted via β-elimination to crotonic acid by heating in concentrated sulfuric acid; crotonic acid may then be isolated and assessed by high pressure liquid chromatography [21,45]. The ^1^H-NMR resonances of PHB are distinctive (Figure 5A), but the concentration of cPHB in proteins is very low so that this method requires extensive proteolysis and concentration of the cPHB [46]. Thus far, cPHB has been removed from its complexes with proteins only by treatments which effect hydrolysis of the ester bonds, *i.e.*, heating in dilute acid or refluxing in methanol:chloroform mixtures [14]. cPHB-peptides may also be recognized by MALDI MS [26] (see Figure 6 below).

## 6. cPHB-Proteins in *Escherichia coli*

**Figure 7:**
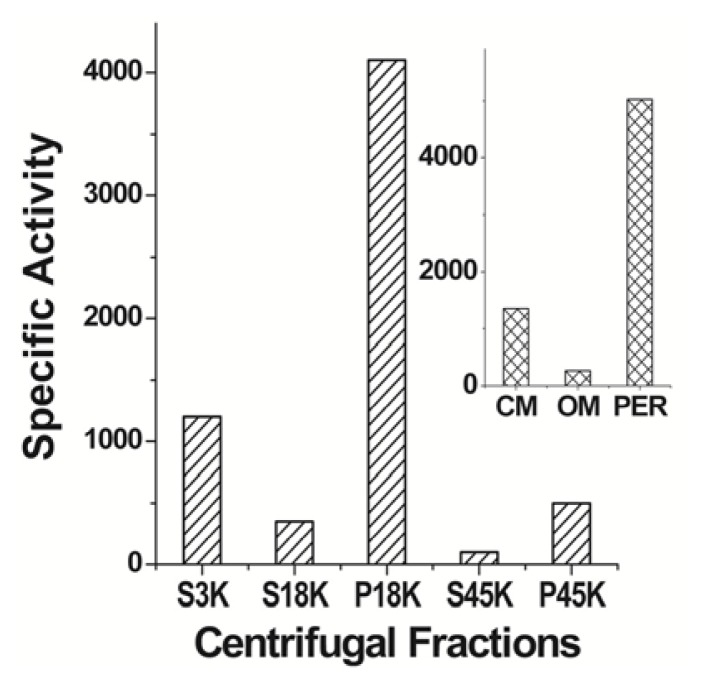
cPHB synthase activity in centrifugal fractions of *E. coli* BW25113 lysates. S, supernatant; P, pellet. Late log-phase cells were lysed by ultrasonication. The low-speed pellet (P3K) was discarded and the supernatant (S3K) was separated into the following density fractions: envelope fraction (P18K), cytoplasm (S18K), ribosomal fraction (P45K), and cytosolic fraction (S45K). PHB synthase activity was assayed in each fraction by following the conversion of water-soluble ^14^C-3-HB-CoA into TCA-insoluble oligomers. Insert shows distribution of activity in P18K fractions (envelope fractions). CM, cytoplasmic membrane; Per, periplasm; OM, outer membrane. Specific activity; counts per minute per mg protein. From Dai and Reusch [47].

cPHB-proteins have been most extensively examined in *E. coli*. It is estimated that cPHB comprises 0.36%–0.55% of the dry weight of log-phase *E. coli* cells [21]. The cPHB-proteins are widely distributed within the cell. Western immunoblots, probed with polyclonal anti-PHB IgG, revealed cPHB-polypeptides in all cell fractions. Surprisingly, the majority of the cPHB-proteins (over 80%) are located in the cytoplasm, with the largest concentration in the ribosomal fraction. The identity of ribosome-associated cPHB has been confirmed by both chemical assay and ^1^H-NMR spectroscopy (Figure 7B) [21]. Some of the cPHB-proteins of *E. coli* that have been identified thus far by two-dimensional gel electrophoresis followed by Western blot analysis with anti-PHB IgG are listed in Table 1.

It has been proposed that two pathways—short-fatty-acid catabolism (atoDAEB operon) and fatty-acid metabolism—participate in the synthesis of cPHB in *E. coli* through the involvement of the AtoSC signal-transduction system [48–50]. Rychlewski *et al.* [51] used structural similarity with known polymerases and their characteristic GXCXG sequence to identify three potential cPHB polymerases in *E. coli*—a cytoplasmic polymerase, ysgA, and two other polymerases of unknown cellular location, ycjY and yghX. Dai and Reusch [47] found that cPHB polymerase activity is particularly strong in the periplasm, which displays ~75% of the total activity (Figure 7). They identified one periplasmic cPHB polymerase as ydcS, a putative periplasmic-binding protein of an ABC transporter system, ydcSTUV [52]. The outer membrane of a ydcS deletion mutant of *E. coli* contained ~30% less cPHB than the wild-type protein. The primary structure of ydcS suggests that it may be a member of the α/β hydrolase superfamily of proteins [53], which includes class III polyhydroxyalkanoates polymerases as well as lipases and esterases [54].

## 7. The Role of cPHB in Folding of Outer Membrane Protein A (OmpA) of *Escherichia coli*

The physical properties and distribution of cPHB imply that it plays important roles in protein structure and physiology. The best example to-date of the influence of cPHB on protein targeting and protein folding is the outer membrane protein A (OmpA) of *E. coli*. OmpA is a major protein of the cell envelope with many functions [55]. It stabilizes the outer membrane [56], acts as a receptor for bacteriophages [57,58], participates in bacterial conjugation [59,60], is a target in the immune response [61], mediates virulence and pathogenicity [62–65] and participates in biofilm formation [66]. In addition, OmpA has served as a paradigm for studies of the biogenesis of outer membrane proteins [67].

### 7.1. Structure of OmpA

The mature conformation of OmpA has been a matter of contention for several decades. A narrow-pore, two-domain structure in which 171 *N*-terminal residues form a narrow eight β-barrel pore in the outer membrane and 154 *C*-terminal residues form a globular structure that interacts with peptidoglycan in the periplasm has vied with a large-pore, single-domain structure in which all or most of the 325 residues create a large pore in the outer membrane.

A two-domain conformation of OmpA was proposed based on bacteriophage mapping [57,58], proteolysis [68,69], and mutagenesis studies [70,71]. The structure of the narrow pore formed by the *N*-terminal domain—residues 1–171—has been described by fluorescence, circular dichroism, Raman and NMR spectroscopy [72–75], by X-ray crystallography [76,77], and by molecular simulations [78,79]. The results of these studies indicate that the *N*-terminal domain forms an antiparallel eight-stranded-β-barrel connected by long, unstructured and mobile loops at the extracellular side and short, tight loops at the periplasmic side. The interior of the β-barrel, lined with charged and polar residues, encloses several unconnected aqueous cavities. The *C*-terminal domain has been crystallized [80,81] and circular dichroism studies indicate that this domain adopts a mixed alpha/beta secondary structure [82].

The structure of the single-domain large pore is yet unknown; however, a large pore structure is consistent with the known functions of OmpA as a receptor for bacteriophages [57,58] and participant in bacterial conjugation [59,60], both of which imply it forms a pore large enough for the passage of ssDNA. Involvement of the *C*-terminal domain in formation of a large pore is further indicated by studies of OmpA homologues, *Pseudomonas aeruginosa* OprF by Rawling *et al.* [83] and *Salmonella enterica* OmpA (94% identical to *E. coli* OmpA) by Singh *et al.* [84], in which monoclonal antibodies were used to prove that *C*-terminal epitopes of the proteins were exposed on the cell surface of intact bacteria.

**Figure 8:**
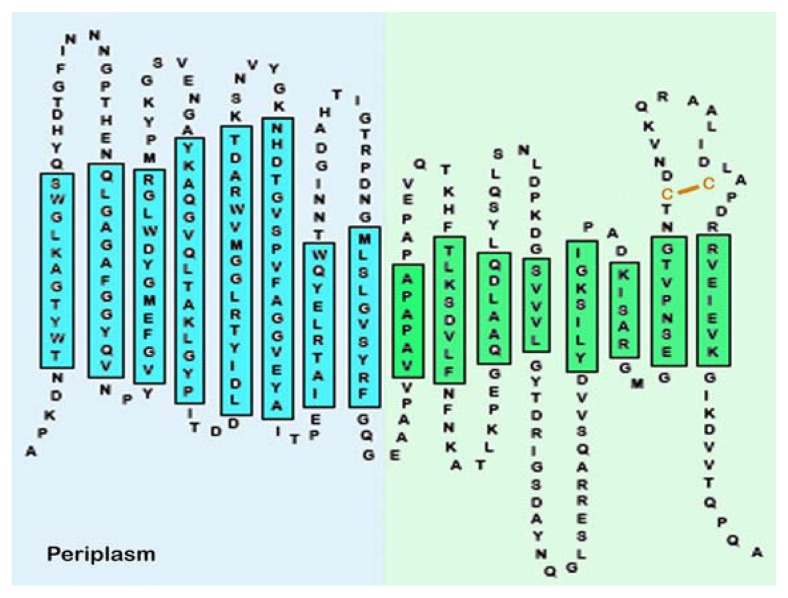
Composite topological structure of OmpA. Left (blue): topological of the *N*-terminal domain of OmpA (residues 1–175), as determined by X-ray crystallography performed by Pautsch & Schultz [76,77]. Right (green): hypothetical topology of the *C*-terminal domain (residues 175–325), as proposed by Stathopoulos [88]. From Reusch 2012 [94].

Computer modeling studies have supported the involvement of the *C*-terminal domain in pore formation. Jeanteur *et al.* [85] predicted two transmembrane β—strands within the *C*-terminal domain, and additional *C*-terminal transmembrane segments were predicted by algorithms of Schirmer and Cowan [86] and Ferenci [87]. Stathapoulos [88] constructed a 16 β-barrel structure with eight transmembrane segments in the *C*-terminal domain that is consistent with the varied biochemical, immunological, and genetic topological data concerning OmpA (Figure 8).

Experimental evidence for a large pore structure for OmpA is provided by studies of Sugawara and Nikaido [89,90] which showed that 2%–3% of the molecules form nonspecific diffusion pores of ~1 nm diameter in liposomes and planar bilayers, and by studies of Arora *et al.* [91] in which two interconvertible conductance states were observed: a major population of small pores (50–80 pS) and a minor population of large pores (260–320 pS). The large pore conformer was not observed when only the *N*-terminal domain was present, which supports the premise that the *N*-terminal membrane portion of OmpA is sufficient for formation of the small channel whereas the large channels require the presence of both domains. The existence of a large pore structure is further supported by planar lipid bilayer studies of OmpA by Zakharian and Reusch [92,93], which show that large pores (~450 pS) are the dominant structures (open probability of 0.9) at physiological temperature of 37 °C (see below).

### 7.2. Targeting and Folding of OmpA

OmpA must be transported from the ribosome to the cytoplasmic membrane, conveyed across the cytoplasmic membrane into the periplasm, conducted across the periplasm, inserted into the outer membrane and folded therein in its mature conformation. During this journey, modifications may be made to the protein by cytoplasmic and/or periplasmic enzymes. Studies of OmpA structure have been performed with OmpA obtained by protein overexpression into cytoplasmic inclusion granules or with OmpA directly extracted from the outer membranes of *E. coli*. In both cases, OmpA will have acquired modification(s) made to the protein in the cytoplasm but only OmpA extracted from the outer membranes will have acquired any additional modification(s) that may be made in the periplasm.

#### 7.2.1. From the Ribosome to the Cytoplasmic Membrane

The OmpA precursor (ProOmpA) is synthesized in the cytoplasm as a 325-residue protein with a 21-residue signal sequence at the amino terminal. Studies by the Henning group [95–99] have demonstrated that residues 163–170 (SLGVSYRF) of the *N*-terminal domain of OmpA, known as the sorting signal, are essential for the integration of OmpA into the outer membrane. When the sorting signal is removed, OmpA remains in the periplasm. Xian *et al.* [26] established by Western blot immunoassay and chemical assay that both serine residues in segment 162–174 (LSLGVSYRFGQGE) of OmpA are modified by cPHB. The studies further indicated that the serines are modified only when bordered by hydrophobic residues. In the S167G mutant, replacement of either of the hydrophobic residues adjacent to S163–L162 or L164—with glycine prevented cPHB-modification of S163. Similarly, in the S163G mutant, replacement of the hydrophobic residue V166 by glycine inhibited cPHB-modification of S167. Segment 162–174 was modified by cPHB in protein isolated from cytoplasmic inclusion bodies as well as in protein isolated from outer membranes, indicating that the attachment of cPHB takes place in the cytoplasm. The cPHB could not be removed from peptide 162–174 by solvent extraction, suggesting it is covalently attached to the serines at its CoA ester end.

cPHB is not discernible in the Raman or ^1^H-NMR spectra [73–75] or X-ray structures [76,77] of the *N*-terminal domains. Due to its low concentrations and highly flexible backbone, cPHB would resemble detergent or lipid molecules in these spectra. However, the modification of OmpA peptide 162–174 by up to 10 R-3HB residues is visible in the MALDI-MS spectra [26] (Figure 6). The lability of the ester bonds of cPHB is indicated by differences between the upper and lower spectra. In the upper spectrum, the molecular ion (1412) is buried in the noise but strong peaks are observed that correspond to covalent attachment of 10, 8, and 6 units of R-3HB. In the lower spectrum, obtained after a few additional seconds of exposure of the sample to the laser beam, the molecular ion has become visible, while the peak attributed to covalent attachment of R-3HB of 10 units has diminished, and peaks attributed to covalent attachment of R-3HB of 8 and 6 units have intensified. The R-3HB units appear to be degraded by the laser in pairs and fewer than 6 R-3HB units were not observed. It is interesting to note that the enzymatic degradation of storage PHB also proceeds with the loss of R-3HB dimers [100]. Continued exposure to the laser beam ultimately results in completely degrading the cPHB. The modification of the serines in segment 162–174 by cPHB is further indicated by MALDI-MS of the double mutant S163G/S167G, which showed a strong peak for the molecular ion and no higher molecular weight peaks attributable to modification by cPHB [26].

**Figure 9:**
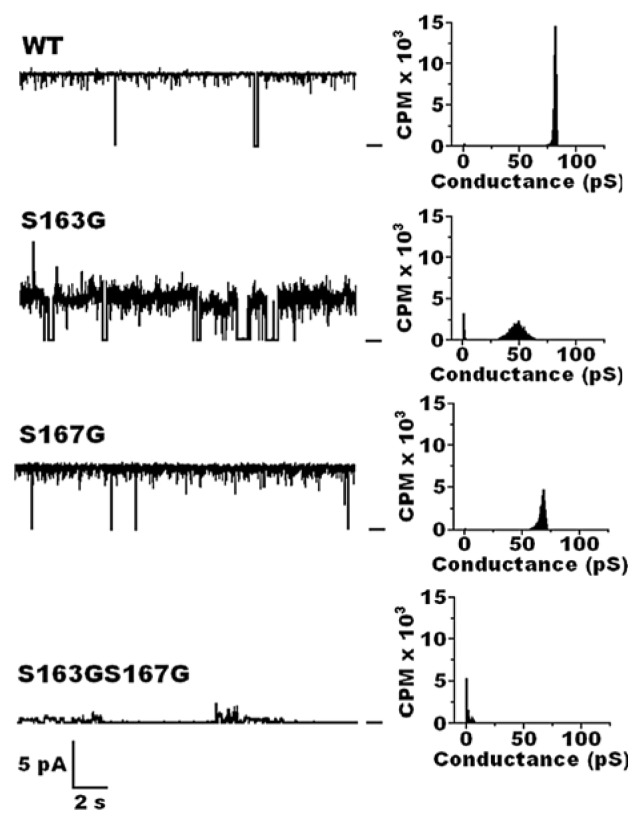
(**Left**) Representative current traces of OmpA wild-type and mutant OmpA proteins isolated from cytoplasmic inclusion bodies of *E. coli* BL21(DE3) pLysS cells The proteins were incorporated into planar lipid bilayers of 1,2-diphytanoyl-*sn*-glycero-3-phosphocholine (DPhPC) at room temperature between aqueous solutions of 1 M KCl in 20 mM HEPES, pH 7.4. Bar at right of the traces indicates the closed state of the channel. Clamping potential was +100 mV with respect to ground; (**Right**) Histograms taken from records of *ca.* 10 min at room temperature. From Negoda *et al.* BBA 2010 [101].

Negoda *et al.* [101] demonstrated the importance of cPHB modification of the sorting signal to the incorporation of OmpA into planar lipid bilayers as a narrow pore at room temperatures (Figure 9). Wild-type OmpA (WT) formed well-structured low-conductance narrow pores (80 ± 2 pS) with high open time (~0.9). Single serine mutants S163G and S167G, which still contain some cPHB on the sorting signal, retained the ability to form narrow pores in planar lipid bilayers; however, the partial loss of cPHB resulted in significantly lower conductance and open time. The double mutants, S163G/S167G and S163V/S167G, which are completely without cPHB on the sorting signal, displayed only trial insertions into the bilayer, which suggests that these mutant proteins are associated with the bilayer and may even lie within it, but are unable to form stable pores. Clearly, the attachment of cPHB to hydrophilic serine residues in the eighth β-strand decreases the hydrophilicity of this segment and thereby assists in targeting the protein to the bilayer and/or inserting the protein into the bilayer as a narrow pore.

#### 7.2.2. Across the Periplasm to the Outer Membrane

After translocation across the cytoplasmic membrane, the signal sequence is cleaved and OmpA, assisted by periplasmic chaperones and lipopolysaccharides, is inserted into the outer membrane and folded into its native form [102–105]. In order to form the large-pore structure, hydrophilic segments of the *C*-terminal domain must insert into the outer membrane bilayer, and this requires an increase in their hydrophobicity. This may be accomplished by the attachment of cPHB to specific amino acids.

Modification of the *C*-terminal domain by cPHB is a decisive event in the translocation and folding of OmpA into the outer membrane in its mature large-pore conformation. Studies by Negoda *et al* [46]. demonstrated that the *C*-terminal domain of OmpA 264–325 is modified by cPHB and that this modification takes place in the periplasm, *i.e.*, Western Blot and chemical assays indicated that segment 264–325 contains cPHB when OmpA is isolated from outer membranes, but not when OmpA is isolated from cytoplasmic inclusion bodies. The identity of cPHB on segment 264–325 of OmpA was confirmed by ^1^H-NMR. The increased hydrophobicity conferred to this segment by addition of cPHB is indicated by the solubility of segment 264–325 in chloroform when it is obtained from outer membranes, in contrast to its insolubility in chloroform when it is obtained from inclusion granules. The specific residues that are modified by cPHB within this segment have not been identified. It is also unknown whether other segments of the *C*-terminal domain are modified by cPHB.

**Figure 10:**
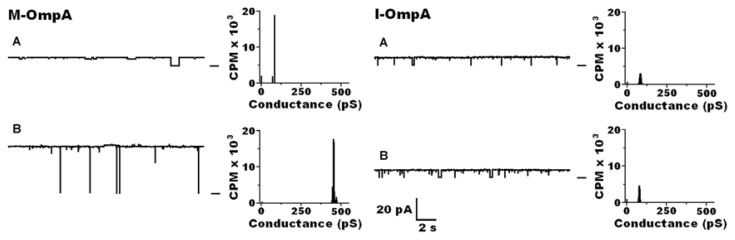
Representative single-channel current traces of OmpA extracted from outer membranes (M-OmpA) and OmpA extracted from inclusion bodies (I-OmpA) *in E. coli* BL21(DE3)pLysS cells. M-OmpA. Each protein was isolated with LDS, reconstituted in C8E4 micelles, and incorporated into bilayers of 1,2-diphytanoyl-*sn*-glycero-3-phosphocholine (DPhPC) between aqueous solutions of 20 mM Hepes (pH 7.4) and 1 M KCl at 22 °C. (**A**) M-OmpA and I-OmpA at 22 °C; (**B**) M-OmpA and I-OmpA at 22 °C after incubation at 40 °C for 2 h. The closed state is indicated by the bar at the right of each trace. The clamping potential was +100 mV with respect to ground (trans). The corresponding histograms from 1 min of continuous recording show the distribution of conductance magnitudes. CPM, counts per minute. From Negoda *et al.* FEBS 2010 [46].

Studies by Zakharian and Reusch [92,93] illustrate the importance of temperature in the folding of OmpA into its native large-pore form. They showed that OmpA, whether isolated from outer membranes (M-OmpA) or from inclusion bodies (I-OmpA), forms narrow pores in planar lipid bilayers at room temperatures. However, as temperatures are raised to physiological values, M-OmpA but not I-OmpA undergoes a transition from a narrow low-conductance pore to a large high-conductance pore structure (Figure 10). Further studies [101] indicated that this folding step is steeply temperature-dependent, indicating a high activation energy, estimated as ~139 kJ/mol, which is indicative of a major conformational rearrangement. The temperature-sensitivity of the cPHB backbone likely contributes to this high energy of activation. *In vivo*, the folding process may be assisted by chaperones that lower the activation energy.

**Figure 11:**
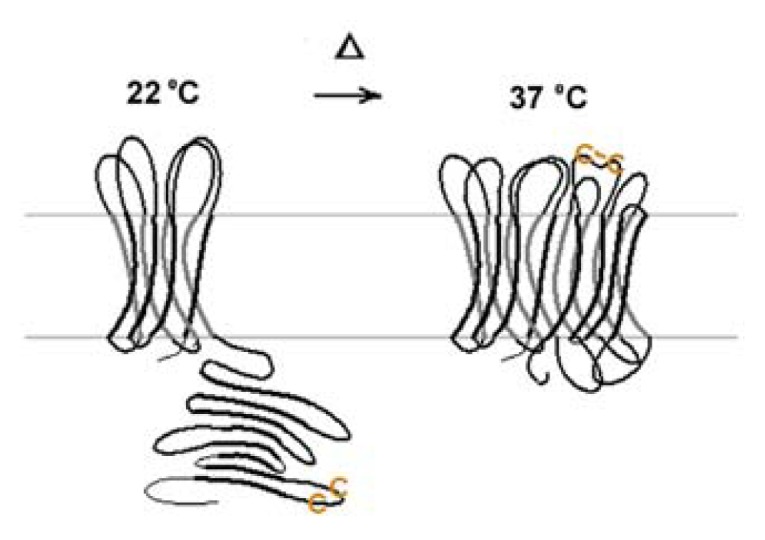
Schematic drawing of the transition from a narrow 8 β-barrel pore at lower temperatures to a large hypothetical 16 β-barrel pore at higher temperatures. From Zakharian and Reusch Biochem 2005 [93].

Once the conformational rearrangement has been completed, the large pore is very stable. The transformation is irreversible. Conversion of large pores to narrow pores with decreasing temperature was not observed. Even extended periods at low temperatures did not reverse the transition. Large pores are converted to narrow pores only when they are subjected to denaturing conditions. The sum of the data indicates strongly that the mature large pore structure is the end-product of the folding cascade (Figure 11).

**Figure 12:**
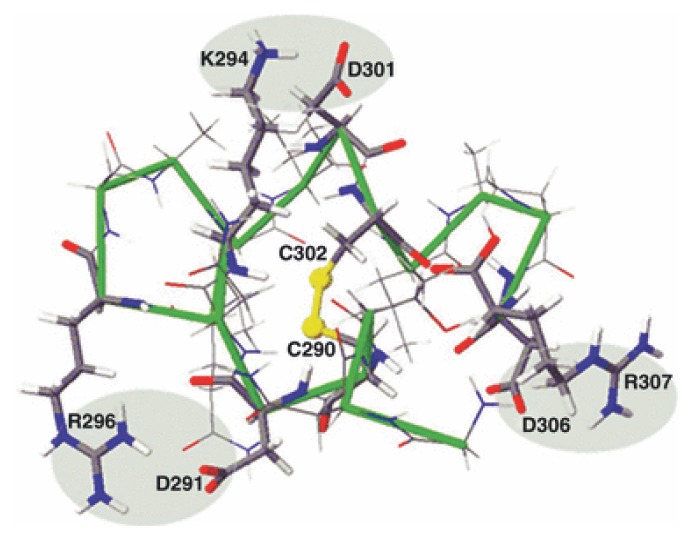
Molecular model of the longest extracellular loop formed by residues 288–307 of the *C*-terminal domain of OmpA [46]. Red: positive residues. Blue: negative residues. Yellow: Cys residues. The backbone is traced in green. Salt bridges are shown in gray ovals.

Although cPHB-modification is an effective process for increasing the hydrophobicity of polypeptide segments destined to remain within the bilayer, it may not be suitable for polar segments of the *C*-terminal domain that must traverse the bilayer to reach the extracellular aqueous medium. Another periplasmic modification that is also critical to the proper folding of OmpA is the formation of a disulfide bond between Cys290 and Cys302 of the *C*-terminal domain [46] by the oxidizing protein DsbA [106,107]. This modification clearly plays no role in the formation of the narrow pore by the *N*-terminal domain and thus it was considered to be irrelevant to the proper folding of the protein. In the Stathopoulos model of OmpA [90], residues 288–307 form a long extracellular loop (Figure 8). In addition to the Cys residues, this segment includes six charged residues (three positive and three negative). Molecular modeling studies by Negoda *et al.* [46] suggest that formation of a Cys290–Cys302 disulfide bond may facilitate bilayer transfer of this putative segment by enabling the formation of salt bridges between the oppositely charged residues and packaging it into a more compact structure (Figure 12). This conjecture is supported by planar bilayer studies [46], which showed that the Cys290–Cys302 disulfide bond was essential for OmpA to undergo the narrow-pore to large-pore transition, but it was not essential for retaining the large pore conformation once the transition has been completed.

From these studies, one may infer that the native structure of OmpA in *E. coli* is that of a single-domain large membrane pore, and that the two-domain structure is a folding intermediate that is unusually stable at lower temperatures. It is clear that cPHB-modification is essential to the formation of both the narrow pore and large pore conformations. Without cPHB modification of the *N*-terminal domain of OmpA in the cytoplasm, the narrow pore either does not form or it cannot fully insert into the bilayer; without cPHB modification of the *C*-terminal domain and creation of the Cys290–Cys302 disulfide bond in the periplasm, the large pore does not form. Studies of OmpA, which concluded that the native structure is a two-domain narrow pore, obtained the protein by overexpression [67,72–75,102,103], and thus it was lacking vital periplasmic modifications. Studies of OmpA, in which the large pore was observed, but only as a minor conformer, acquired the protein from the outer membranes but it was maintained at or below room temperatures [89,90] or it was damaged by exposure to urea at elevated temperatures [91]. In summary, the folding of OmpA into its native structure requires that the protein undergo cPHB-modifications in both the cytoplasm and periplasm, disulfide bond formation in the periplasm AND, due to the temperature sensitivity of the cPHB backbone, it also requires physiological temperatures.

## 8. Concluding Remarks

cPHB constitutes yet another class of protein conjugates. This polyester is a ubiquitous constituent of both prokaryotic and eukaryotic cells in which it is primarily found covalently attached to proteins located within membranes and organelles. cPHB possesses physical properties that enable it to play important roles in the targeting, folding and function of these proteins. Clearly, the prospect of cPHB-modification should always be considered when designing protocols for isolating and refolding proteins for structural studies. Protein overexpression is a convenient method of obtaining ample amounts of protein for spectroscopic and crystallographic studies, but a functional protein is more than a chain of amino acids. Posttranslational modifiers of the protein should be identified and their potential roles in attaining the native structure carefully evaluated. In addition, it is important to realize that although the polypeptide structure of the protein is not strongly temperature-dependent, modifiers such as lipids and cPHB may make it so. Unless all temperature-dependent modifiers are ruled out, protein folding is best observed within the temperature range at which it occurs in nature.

## Figures and Tables

**Table 1:** cPHB proteins in *E. coli* lysates were identified by two-dimensional electrophoresis followed by Western blot analysis using anti-PHB IgG. OM—outer membrane; CM—cytoplasmic membrane.

Protein	MW kDa	Gene	Cellular location
OmpA	37.2	*ompA*	OM
OmpF	39.3	*ompF*	OM
OmpC	40.3	*ompC*	OM
OmpW	22.9	*yciD*	OM
Braun’s Lipoprotein	7.2	*lpp*	OM
ATP synthase, β subunit	50.3	*atpD*	CM
GroEL	57.1	*mopA*	CM
EF-Tu	43.2	*tufA*, *tufB*	CM
EF-TS	30.2	*tsf*	Cytoplasm
DNAK	69.0	*dnaK*	Cytoplasm
L7/L12	12.2	*rplL*	Cytoplasm
S1	61.1	*rpsA*	Cytoplasm
H-NS	15.4	*hns*	Cytoplasm
RNA polymerase, α subunit	150.6	*rpoA*	Cytoplasm
